# Pathological Waltz

**Published:** 1831

**Authors:** 


### LXVII.

### Pathological Waltz.

Dr. Corrigan, Dr. Hope, and many others will be delighted to learn that Dr. David Badham has set to music the discordant sounds of a diseased heart, and has thus drawn harmony out of hypertrophy, with more than Paganini's skill. The bars, crotchets, quavers, and demiquavers are tunefully arranged in the pages of an esteemed contemporary, and form one of the greatest curiosities in morbid anatomy which we have ever witnessed. The case was that of a young woman who was treated in the wards of the Royal Infirmary of Glasgow, for pleurisy, and was "completely recovered," and about to be discharged, when it was discovered that her recovery was very far indeed from being so complete. Her respiration was never perfectly easy, and she was put out of breath by the slightest exertion. Her pulse was very uncertain as to frequency, varying from 45 to 115 in the minute. But the following very curious results of various examinations gave origin to the musical, and we would say, very fanciful illustration, to which we have just adverted.

"1. Pulsation of the heart heard over the whole chest, or nearly so.

2. The heart's impulse on the right side exaggerated, but slightly; neither the first nor second sound, however, so clear as on the opposite side, where,

3. The former is far louder than natural, but the shock less than over the right cardiac region.

4. The first sound concomitant with the heart's impulse is preternaturally loud on both sides, more particularly on the left, where it resembles the sound commonly produced by the auricles.

5. The rhythm of the heart has been for the last week or ten days altogether extraordinary, and to this fact my attention has been particularly drawn; the first sound, with its concomitant impulse, having been as usual followed immediately by a second; this was in its turn as immediately succeeded by a third, or, to speak more correctly, by a reduplication of the second. These three sounds together form an exact *waltz* measure, after which comes a pause of much longer than natural persistence. On some occasions the auricle (to which I have little hesitation in referring the second sound of the heart, although at variance, perhaps, with the prevailing opinion) has contracted not only twice, but sometimes thrice, or even four times, for each pulsation of the ventricle; and what is very remarkable, since contractions of the auricles are seldom communicated to the sense of touch, distinct beats exactly synchronous with these sounds are to be ascertained, whatever number of times the sounds are repeated. In short, if the ventricles in this case occupied the same time in contracting as they do in health, and the pause were of natural persistence, the rhythm of the heart might be represented in musical notation, as follows:"—

[Here follows the music, which we had not time to represent by wood-cuts, and therefore the reader must take the verbal description.]

"The first bar represents the natural rhythm, in which the ventricle occupies half of the time, the auricle quarter of the time, and the pause the remaining quarter."

The irregularity in question did not escape the observation of Laennec.

"It sometimes (says Laennec) though very rarely, happens during palpitation, that each contraction of the ventricle is followed by several successive contractions of the auricles, so quick as only to equal, in point of time, one ordinary contraction. In this sort of palpitation, I have sometimes reckoned two pulsations of the auricles for one of the ventricles—sometimes four, but more commonly three."

In addition to the above phenomena, the patient of Dr. B. presents a widely diffused "bruit de soufflet" down the course of the aorta, and throughout its extent. Dr. B.'s diagnosis is, "dilatation with slight hypertrophy of the right, and extensive passive aneurism of the left side of the heart."

---

